# Modal Structuralism with Theoretical Terms

**DOI:** 10.1007/s10670-021-00378-w

**Published:** 2021-05-08

**Authors:** Holger Andreas, Georg Schiemer

**Affiliations:** 1 Department of Economic, Philosophy, and Political Science, University of British Columbia (Okanagan), Kelowna, Canada; 2 Department of Philosophy, University of Vienna, Vienna, Austria

## Abstract

In this paper, we aim to explore connections between a Carnapian semantics of theoretical terms and an eliminative structuralist approach in the philosophy of mathematics. Specifically, we will interpret the language of Peano arithmetic by applying the modal semantics of theoretical terms introduced in Andreas (Synthese 174(3):367–383, 2010). We will thereby show that the application to Peano arithmetic yields a formal semantics of universal structuralism, i.e., the view that ordinary mathematical statements in arithmetic express general claims about all admissible interpretations of the Peano axioms. Moreover, we compare this application with the modal structuralism by Hellman (Mathematics without numbers: towards a modal-structural interpretation. Oxford University Press: Oxford, 1989), arguing that it provides us with an easier epistemology of statements in arithmetic.

## Introduction

Logical atomism is a key semantic doctrine in Russell’s conception of analytic philosophy (Russell 2010). It was adopted by his student Wittgenstein in the *Tractatus Logico-Philosophicus* (1922), which is centred on the notion of an elementary sentence. Prior to Russell, we can recognize the spirit of logical atomism in Frege’s work on the foundations of arithmetic. Frege attempted to explain the notion of natural numbers in such a manner that he could say what a specific natural number is, such as the number referred to by the numeral “7”. The natural numbers were construed as specific second-order concepts, the extension of which hinges on the truth values of first-order atoms.

Not surprisingly, Carnap’s first major work, *The Logical Structure of the World* (*Der Logische Aufbau der Welt*) (1928), was very much guided by the semantic principle in question. There, the atomic sentences were given by sentences that assert whether or not two elementary experiences are similar. All further concepts concerning the spatiotemporal world—except for the relation of temporal order—had to be introduced via rigorous definitions in the logical system of the *Principia Mathematica* by Russell and Whitehead (1925).

Carnap, however, deviated from the principle of logical atomism not too long after the *Aufbau * appeared. This deviation culminated in a semantics of theoretical terms that is centred on the doctrines of indirect and incomplete interpretation (Carnap 1958). On this semantics, the axioms of a scientific theory *T* are *postulates* that have a twofold function: first, determining the meaning of *T*’s theoretical terms, and second, setting forth the empirical content of *T*. Hence, unlike in the frameworks adherent to logical atomism, we cannot always clearly distinguish between making an assertion about the world and specifying the meaning of a concept. It is only at the level of the whole theory—but not at the level of single axioms—that we can draw this distinction (Carnap 1958).

In some strands in the philosophy of mathematics, we can observe a similar deviation from logical atomism. Benacerraf’s seminal “What Numbers Could not Be” (1965) can be read as a demonstration that it is mistaken to seek for unique referents of our numerals, and to identify these referents as the natural numbers. It is therefore impossible to recognize certain atomic units as semantic foundations of arithmetic. Rather, it is numerous sequences of objects that allow us to verify (model-theoretically) the propositions of arithmetic, and we identify these sequences using the Peano axioms or a set of axioms that entail these axioms. This insight gave rise to structuralist philosophy of mathematics.

Various types of mathematical structuralism have been developed in the wake of Benacerraf (1965), some of which are formally elaborated. In particular, we distinguish between forms of *eliminative* and *non-eliminative* structuralism. Non-eliminative structuralists take mathematicians’ talk of structures at face value. According to them, there exists a natural number structure as the subject matter of arithmetic in addition to the set-theoretic number systems satisfying the Peano axioms. Shapiro’s theory of *ante rem* structures in Shapiro (1997) presents the best-known version of such a non-eliminative approach. Ironically, we can view ante-rem structuralism as a conservative attempt at restoring the doctrine of logical atomism in the face of arithmetic apparently lacking a unique interpretation.

Types of eliminative structuralism, by contrast, are characterized by the view that mathematician’s talk of structures is just an economical means to paraphrase talk about *all* set-theoretic systems of a theory. Structures, in this view, do not exist as abstract entities in addition to these systems. For instance, Peano arithmetic does not have a natural number structure as its subject matter, but it can be interpreted in infinitely many set-theoretic systems satisfying the Peano axioms. Hellman’s modal structuralism given in Hellman (1989) presents the most formally developed theory of eliminative structuralism for arithmetic and set theory. Hellman’s account presents a direct elaboration of Putnam’s “modal picture” of mathematics first outlined in Putnam (1967). Notably, Putnam’s modalism presents a similar deviation from the logical (or set-theoretic) atomism in mathematics as the one articulated by Benacerraf in 1965.

In this paper, we aim to connect the two lines of research on alternatives to logical atomism. That is, we aim to explore connections between a Carnapian semantics of theoretical terms and an eliminative structuralist approach in the philosophy of mathematics. Specifically, we will interpret the language of Peano arithmetic by applying the modal semantics of theoretical terms introduced in Andreas (2010). This semantics has been devised to capture Carnapian ideas about an indirect and incomplete interpretation of theoretical terms in Carnap (1958). We will thereby show that the application to Peano arithmetic yields a formal semantics of universal structuralism, i.e., the view that ordinary mathematical statements in arithmetic express general claims about all admissible interpretations of the Peano axioms (cf. Schiemer and Gratzl 2016). Moreover, we compare this application with the modal structuralism developed in Hellman (1989), arguing that it provides us with an easier epistemology of statements in arithmetic.

## Theoretical and Observational Terms

What is a theoretical term? There are at least two ways to explain the notion of such a term. First, a theoretical term is one that is not part of our observation language. Second, a theoretical term semantically depends on some theory. These explanations require further explications. What is it for an expression to be part of our observation language? What does it mean that a term semantically depends on a theory? Let us begin with answering the first question.

In Carnap (1936/37) and Carnap (1966, Ch. 23), we find some informal explanation of what an observation term is. In essence, a term belongs to the observation language if and only if it can be applied without using a theory or artefacts. Carnap emphasizes that the theory-observation distinction is not clear-cut so that, when formalizing a scientific theory, we introduce a sharp line where there is in fact a continuum (see Carnap 1936/37, p. 455 and Carnap 1966, p. 226). A physicist has a wider understanding of observability, which includes quantities for which there is some straightforward measurement method available. For example, a physicist views the concept of temperature as observable, at least for medium-sized objects and within a certain temperature range. A philosopher, by contrast, is interested in the semantic foundations of even relatively straightforward measurement methods. For the philosopher, temperature is theoretical.

Sneed (1979) and Balzer et al. (1987) tried to sharpen the theory-observation distinction by adopting a relativized criterion of theoreticity. Roughly, they suggested the following criterion: a term *t* is theoretical—relative to theory *T*—if and only if any method of determining the extension of *t* requires some axiom of *T*. Lewis’ (1970) distinction between old and new terms, the latter being introduced by some new theory *T*, is driven by similar ideas. Note that the criterion by Sneed (1979) and Balzer et al. (1987) gives us an explanation of what it is for a term to semantically depend on a theory. Standard examples of this type of theoreticity are the force and the mass function in classical mechanics. Suppose we construe the notion of classical mechanics broadly so that it includes collision mechanics, Hooke’s law, Newton’s law of gravitation and a few further laws. Then, it holds that all methods for determining the (classical) force acting upon an object and all methods for determining the (classical) mass of an object rest on some axiom of classical mechanics. That is, any method that allows us to measure either of these functions is valid only if we accept some axiom of classical mechanics (see (Balzer et al. 1987, Ch. 3) for details). A case in point is using a coil spring in order to determine the gravitational force on a medium-sized object nearby the earth’s surface. This measurement method rests on Hooke’s law.

One problem with the relativized criterion of theoreticity is that we often have different measurement methods for one and the same concept. For example, we can measure the temperature of a liquid using a gas thermometer, a mercury thermometer, a resistance thermometer, and a few more types of thermometers. The working of these thermometers is based on different theories so that each measurement method semantically depends on a specific theory. The gas thermometer, for examples, exploits the kinetic theory of heat and the ideal gas law. The resistance thermometer, by contrast, is based on other theories, namely theories of electrical conductivity and Brownian theories of motion for solid objects. This, however, implies that there is no single theory such that any method for measuring temperature depends on this theory. The relativized criterion by Balzer et al. (1987), therefore, yields that temperature is not theoretical relative to any specific scientific theory, such as the kinetic theory of heat. This conclusion is counterintuitive. To avoid the conclusion, we understand theoreticity relative to a set of theories. That is, a term *t* is theoretical—relative to a set ${\mathbf {T}}$ of theories—if and only if (i) any method for determining the extension of *t* is based on some member of ${\mathbf {T}}$, and (ii) each member of ${\mathbf {T}}$ justifies some method for determining *t*. We shall adopt this variant of a relativized criterion of theoreticity for our discussion of the language of the Peano axioms in Sect. 4.

## Modal Semantics of Theoretical Terms

The modal semantics of theoretical terms emerges from Ramsey (1950) and Carnap (1939, 1958). Let us therefore review briefly the basic ideas of the Ramsey account of scientific theories, and then Carnap’s semantics of theoretical terms. According to Ramsey (1950, p. 231), theoretical terms lack a distinct intension, or meaning. Such terms are merely extensionally specified through the context of a corresponding scientific theory, but this specification need not result in a unique interpretation of theoretical terms. As a consequence of this, a theoretical sentence by itself does not have a complete meaning. The meaning and interpretation of such a sentence rather hinges on the context of a corresponding scientific theory.

To explain Ramsey’s formal elaboration of these semantic theses, a few notational conventions are needed. Let $V_o$ designate the set of observation terms and $V_t$ the set of theoretical terms. $L(V_o)$ and $L(V_o, V_T)$ designate the respective languages. Both Carnap and Ramsey distinguish between two types of axioms containing theoretical terms. First, T-postulates, which contain theoretical terms, but no observational terms. Second, C-postulates, which have occurrences of both theoretical and observational terms.^1^ The latter postulates establish connections between the theoretical and the observational vocabulary of the respective scientific theory. Let $P_{TC}$ denote the set of T- and C-postulates. *TC*, by contrast, designates some conjunction of all *T*- and *C*-postulates.

The Ramsey sentence of a theory $P_{TC}$ in the language $L(V_o,V_t)$ is obtained by the following two transformations of *TC*. First, replace all theoretical symbols in this conjunction with variables of appropriate type. Then, bind these variables by existential quantifiers. As result one obtains a sentence of the following form:

$$\begin{aligned} \exists X_1 \ldots \exists X_n TC(n_1, \ldots , n_k, X_1, \ldots , X_n)\qquad \qquad \qquad \qquad (TC^R) \end{aligned}$$

where $X_1, \ldots , X_n$ are higher-order variables, provided $V_t$ does not contain individual constants at the theoretical level. For simplicity, we assume in the remainder of this section that all theoretical terms are relations, as is standard. The Ramsey sentence $TC^R$ says that there is an extensional interpretation of the theoretical terms such that $P_{TC}$ comes out true, in the context of a given interpretation of the observation terms.

Further, the assertion of a theoretical sentence $\phi$ not in $P_{TC}$ needs to be understood in the context of $P_{TC}$. Hence, such an assertion has to be expressed by putting $\phi$ into the scope of the quantifiers of the Ramsey sentence. More precisely, Ramsey (1950, p. 231) seems to give the following instruction of how to translate a theoretical sentence $\phi$ into a corresponding sentence in the Ramsey view:

1 $$\begin{aligned} \begin{aligned} \quad&\exists X_1 \ldots \exists X_n (TC(n_1, \ldots , n_k, X_1, \ldots , X_n) \wedge \phi _{a}([X_1/t_1], \ldots , \\&[X_n/t_n]) \wedge \phi ([X_1/t_1], \ldots , [X_n/t_n])) \end{aligned} \end{aligned}$$

where $$\phi _a$$ stands for the conjunction of sentences that are already affirmed in the language, in addition to the axioms of *TC*. $$\phi _a([X_1/t_1],$$ $$\ldots , [X_n/t_n])$$ stands for the formula that is obtained by replacing every occurrence of a theoretical term in $$\phi _a$$ with its corresponding higher-order variable. Likewise, $$\phi ([X_1/t_1], \ldots ,$$ $$[X_n/t_n])$$ is obtained through this procedure of replacing theoretical terms with higher-order variables. Henceforth, we shall write $$(TC \wedge \phi _a \wedge \phi )^R$$ to refer to a sentence of the form (1).

Even though $(TC \wedge \phi _a \wedge \phi )^R$ is suggested as a translation of $\phi$, Ramsey notes that this translation does not give us a straightforward semantics of theoretical sentences. We should not say that $\phi$ is true if and only if $(TC \wedge \phi _a \wedge \phi )^R$. For both $(TC \wedge \phi _a \wedge \phi )^R$ and $(TC \wedge \phi _a \wedge \lnot \phi )^R$ may be true. The proposed semantics of theoretical sentences remains informal in the end. Ramsey (1950, p. 231) says that a theoretical sentence $\phi$ means the difference between $(TC \wedge \phi _a \wedge \phi )^R$ and $(TC \wedge \phi _a)^R$.

Carnap develops very similar ideas about the semantics of theoretical terms in his *Foundations of Logic and Mathematics* (1939). The key semantic thesis is that theoretical terms are *indirectly interpreted* through axioms of a scientific theory. An interpretation of a symbol is indirect if and only if it is given by one or several sentences of the object language as opposed to an assignment of an extension or intension in the metalanguage. Sentences indirectly interpreting symbols are to be adopted as axioms in the calculus of the theory in question. Only observation terms are directly interpreted to the effect that only propositions of the observation language have a direct factual content.

Furthermore, in Carnap’s “Beobachtungssprache und theoretische Sprache” (1958), we can recognize the elements of a model-theoretic notion of indirect interpretation. There he proposes to divide a scientific theory *TC* into the Ramsey sentence

$$\begin{aligned} TC^R \end{aligned}$$

and the following conditional:

$$\begin{aligned} TC^R \rightarrow TC. \end{aligned}$$

This conditional became later on labelled the *Carnap sentence* of a scientific theory.

Now, Carnap instructs us to understand the Carnap sentence $TC^R \rightarrow TC$ as follows: if there is an interpretation of the theoretical terms that satisfies *TC* (in the context of the given interpretation of $L(V_o)$), the theoretical terms should be understood as designating such an interpretation. Notably, the Carnap sentence indirectly interprets the theoretical terms as their interpretation is characterized by the condition that $TC^R \rightarrow TC$ is always true. This kind of indirect interpretation gives us some explication of what it means that a theoretical term semantically depends on a scientific theory. Carnap, however, does not address the problem that $TC^R \rightarrow TC$ may not uniquely interpret the theoretical terms. Nor does he consider the case where the Ramsey sentence is false.^2^

Eventually, Andreas (2010) suggests a modal semantics of theoretical terms that accounts for the non-uniqueness of an indirect interpretation of theoretical terms. This semantics centres on the notion of an *admissible structure* of the complete language $L(V_o, V_t)$: an $L(V_o, V_t)$ structure is admissible if and only if it (i) extends the given $L(V_o)$ interpretation to interpret the $V_t$ terms and (ii) satisfies the postulates $P_{TC}$, provided there is such a structure. If there is no such structure, all extensions of $L(V_o)$ that interpret the $V_t$ terms may be considered admissible. $L(V_o, V_t)$ structures may interpret $V_t$ terms in a theoretical domain $D_t$ that expands the observation domain $D_o$. Henceforth, let ${\mathcal {A}}_o$ designate the given, or intended, interpretation of the observation language. $Ext({\mathcal {A}}_o, V_t)$ is the set of structures that interpret $V_t$ such that the interpretation by ${\mathcal {A}}_o$ is extended and the domain $D_o$ of ${\mathcal {A}}_o$ expanded. $V_t$ terms are thus interpreted in a domain $D_t \cup D_o$, where two different members of $Ext({\mathcal {A}}_o, V_t)$ may interpret $V_t$ in two different domains whose intersection is $D_o$.^3^*Mod*(*A*) designates the set of models of *A*, where *A* is a set of sentences. To make these ideas about admissible structures precise (Andreas 2010):

### Definition 1

*Admissible structures* $W_a$ designates the set of $L(V_o, V_t)$ structures that are admissible by way of an interpretation of the $V_t$ symbols through the postulates $P_{TC}$.

$$\begin{aligned} W_a={\left\{ \begin{array}{ll} Mod(P_{TC}) \cap Ext({\mathcal {A}}_o, V_t) \\ &{} \,\, \text {if }Mod(P_{TC}) \cap Ext({\mathcal {A}}_o, V_t)\not = \emptyset \\ Ext({\mathcal {A}}_o, V_t) &{} \,\, \text {if }Mod(P_{TC}) \cap Ext({\mathcal {A}}_o, V_t) = \emptyset . \end{array}\right. } \end{aligned}$$

There being a range of admissible structures, or interpretations, of a language $L(V_o, V_t)$ suggests using a simple *S*5 modal framework: let ${\mathcal {M}}$ be a modal structure such that ${\mathcal {M}}=\langle W_a, R \rangle$ and $R=W_a \times W_a$. Then, we can express the assertion of a sentence $\phi$ of a language $L(V_o, V_t)$ as follows:

$$\begin{aligned} {\mathcal {M}}\models \Box \phi . \end{aligned}$$

Equivalently, we can adopt the following notation for the assertion of $\phi$:

$$\begin{aligned} W_a \models _m \phi . \end{aligned}$$

This notation is intended to say that $\phi$ is true in all structures ${\mathcal {A}} \in W_a$.

This semantics is in line with the Carnap-Ramsey idea that the axioms of a scientific theory work as semantic constraints on the theoretical terms, thereby indirectly interpreting such terms. Moreover, it explicitly takes into account that an indirect interpretation of theoretical terms by $P_{TC}$ may not result in a unique interpretation.

A distinctive merit of the modal semantics is that it is inferentially as powerful as classical logic. It is easy to prove that all classically valid inference rules and axioms remain valid on this semantics. To be more precise:

### Proposition 1

*Let* *A* *be a set of* $L(V_o, V_t)$ *sentences and* $\phi$ *be such a sentence. Further* $\Box A$ *is obtained by prefixing all members of* *A* *with the modal box operator* $\Box$. *Then*, $\Box A \models \Box \phi$ *if and only if* $A \models \phi$.

For proof, see proof of Proposition 3 in Andreas (2010). In line with Ramsey (1950, p. 232), we can say that the indirect interpretation of theoretical terms affects the content of theoretical propositions, but not our reasoning with such propositions. Recall, however, from the above discussion that Ramsey himself did not develop a fully formal notation for theoretical propositions other than the axioms $P_{TC}$. (He merely suggested that $\phi$ means the difference between $(TC \wedge \phi _a \wedge \phi )^R$ and $(TC \wedge \phi _a)^R$). The modal semantics aims to account for both the specific type of content of theoretical propositions and the presumed validity of classical first-order reasoning with such propositions.

Another benefit of the modal semantics is that it stays closer to the surface grammar of scientific reasoning than the Ramsey account. For this semantics does not require replacing theoretical terms by variables and putting all theoretical assertions syntactically in the scope of existential quantifiers, of which at least some are higher-order. To be fair, we can “recover” theoretical terms from the Ramsey sentence $TC^R$ and the Carnap sentence $TC^R \rightarrow TC$ in a straightforward manner, using Modus Ponens. But this recovery hinges on the truth of the Ramsey sentence $TC^R$ and the Carnap sentence $TC^R \rightarrow TC$. The idea, of course, is that the Carnap sentence is always true, while the Ramsey sentence is sometimes false. For the Carnap sentence is an analytic postulate that allows us to understand the meaning of the theoretical terms in the first place.

How do we “know” the Carnap sentence is always true? From the perspective of Carnap’s seminal “Meaning Postulates” (1952), we can say that the Carnap sentence constrains our set of possible worlds. The Carnap sentence must be true in all possible worlds. Therefore, the notion of an analytic postulate already comes with a modal semantics. It is surprising that Carnap himself does not discuss the connection between meaning postulates in Carnap (1952) and the Carnap sentence in Carnap (1958). The modal semantics by Andreas (2010) goes beyond Carnap’s ideas about meaning postulates in suggesting that we should consider a certain set of possible worlds, namely $W_a$, as representation of reality. This implies forgoing the idea of a unique possible world that is actual. Once we have given up this idea, a modal semantics of theoretical sentences—supervaluationist in spirit—suggests itself. The upshot is that some form of modal semantics is needed to capture the surface grammar of scientific reasoning with theoretical postulates.

Note, finally, that the modal semantics just presented applies to definitions and postulates alike. As is standard, we can characterize definitions semantically as constraints that satisfy a certain uniqueness clause: a definition imposes a constraint on the interpretation of a defined symbol such that the extension of the defined symbol is uniquely determined by the interpretation of the undefined symbols for all interpretations of the undefined symbols. Carnapian postulates, by contrast, impose a constraint on the interpretation of theoretical terms that may not be unique. At the same time, the modal semantics of theoretical terms does not exclude that some, or even all postulates, impose a unique constraint such that each postulate explicitly defines a specific term. Likewise, we may have recursive definitions among the postulates. Let us therefore understand the notion of a Carnapian postulate as a generalization of the notion of a definition.^4^

## Application to Arithmetic

In the present section, we will discuss the Carnapian account of indirect interpretation of theoretical terms in the context of mathematics. In particular, we will apply the modal semantics of theoretical sentences to Peano arithmetic, that is, to the semantic evaluation of arithmetical statements. As we will see, this model-theoretic analysis closely captures the structuralist intuitions regarding the nature of arithmetical knowledge first expressed by Benacerraf. More specifically, a central semantical thesis elucidated in Benacerraf (1965) is that the primitive terms of arithmetic are referentially underdetermined. That is, terms such as the numeral ‘2 ’ or the successor function symbol ‘S’ in the signature of the language of Peano arithmetic do not refer to particular entities. Rather, they can be interpreted in (infinitely many) different set-theoretical systems, none of which is privileged over the others. The only constraint these systems have to meet is to satisfy the basic structural properties expressed in the axioms of arithmetic.

As we will see, Benacerraf’s semantic thesis concerning arithmetic (and mathematical languages more generally) closely corresponds to the two cornerstones of a Carnapian semantics of theoretical terms. To recall, (i) theoretical terms are indirectly interpreted by a set of postulates, and (ii) this interpretation may not determine the referents of theoretical terms uniquely. Given this correspondence, how can the modal approach be applied to arithmetic? In other words: how can we explicate truth conditions of arithmetical statements in the modal semantics?

To address these questions, let us first fix some terminology.

### Definition 2

*(Peano arithmetic)* Let ${\mathcal {L}}_{A}$ be the second-order language of arithmetic. $\textsf {PA}_2$, aka *Second-order Peano arithmetic*, is axiomatized as follows:

(PA1) $$S(x) \not = 0$$

(PA2) $$S(x) = S(y) \rightarrow x = y$$

(PA3) $$x \not = 0 \rightarrow \exists y (x =Sy)$$

(PA4) $$\forall X ([X(0) \wedge \forall y (X(y) \rightarrow X(S(y)))] \rightarrow \forall y X(y)).$$

Two points of commentary are in order. Notice first that the signature of ${\mathcal {L}}_{A}$ contains just two non-logical constants, namely the numeral ‘0’ and the unary function symbol ‘*S*’ representing the successor function. Other arithmetical symbols such as the function symbols for addition or multiplication or the numerals ‘2’, ‘54’, etc. can be added by explicit or recursive definitions to ${\mathcal {L}}_{A}$. Second, there is an important difference between the language of arithmetic (and, generally, any mathematical language) and the theoretical languages considered in the Carnapian tradition. The latter are usually two-sorted languages of physics that contain both theoretical *and* observational terms. As we saw, theoretical terms are those interpreted indirectly through the postulates in question. Observational terms, in turn, have a fixed interpretation in the observable world.

How can we apply the theory-observation distinction to the language of Peano arithmetic? Recall that in Sect. 2 we said that a term *t* is theoretical—relative to a set ${\mathbf {T}}$ of theories—if and only if (i) any method for determining the extension of *t* is based on some member of ${\mathbf {T}}$, and (ii) each member of ${\mathbf {T}}$ justifies some method for determining *t*. Now, the crucial question is whether or not we can determine the extension of the numeral ‘0’ and the function symbol ‘S’ without a theory about the natural numbers? To determine the extension of the numeral ‘0’ is to determine the referent of this numeral. What is the referent of the numeral ‘0’? Obviously, we cannot answer this question without having some theory about what the natural numbers are. If we say that the numeral ‘0’ refers to the empty set, we already have some theory about the natural numbers. While van Neumann’s theory and Zermelo’s theory of the natural numbers agree on the interpretation of the numeral ‘0’, these two theories obviously diverge as regards the interpretation of the successor function. If we let the numeral ‘0’ refer to itself, we have yet another theory of the natural numbers. We can certainly apply the numeral ‘0’ at a quotidian level in the sense of determining the cardinality of certain sets, such as the cardinality of the set of elephants in the class room. However, even these simple applications presuppose theories, namely an understanding of natural numbers as cardinal numbers and the comprehension principle of some set theory that allows for Urelements. Note, furthermore, that the semantic dependency of the numeral ‘0’ carries over to the function symbol ‘S’. If we need a theory to determine the referent of ‘0’, we also need a theory to determine the extension of the successor function.

In sum, both the numeral ‘0’ and the successor function ‘S’ are theoretical terms in the sense that we cannot determine their referents without having some theory about the natural numbers. To be more precise, these two expressions are theoretical relative to a range of different theories about the natural numbers, each of which yields different interpretations. Hence, $V_o=\emptyset$.

It seems as if we can infer $V_t = \{0, {\mathsf {S}}\}$ from $V_o=\emptyset$. However, we need to show first that the set of Peano axioms is a member of the set of theories which allow us to determine the referents of the expressions ‘0’ and ‘S’. Are the Peano axioms powerful enough for such a determination? Obviously, these axioms leave open to which objects the expressions ‘0’ and ‘S’ refer. But they allow us to determine the referents of ‘0’ and ‘S’ holistically, albeit not uniquely. For a given interpretation of ‘0’ and ‘S’, they help us decide whether or not this interpretation qualifies as a system of natural numbers. In the context of this, recall from Sect. 3 that the semantics of the Carnap sentence, the Ramsey sentence, and the modal semantics have in common that the theoretical terms are interpreted holistically by a set of axioms, where this interpretation may not be unique. We therefore conclude that the set of Peano axioms is a member of the set of theories that allow us to determine the referents of the expressions ‘0’ and ‘S’, and yet note that the interpretation by the Peano axioms is not unique. Hence, we have $V_t = \{0, {\mathsf {S}}\}$. ${\mathcal {L}}_{A}$ thus contains two theoretical terms, but no observational terms.

Given this, the conjunction of axioms (PA1)-(PA4) can be represented as ${\mathsf {PA}}_{\mathsf {2}}[0, {\mathsf {S}}]$. Moreover, the Ramsey-sentence of ${\mathsf {PA}}_{\mathsf {2}}[0, {\mathsf {S}}]$ is the following sentence expressed in the language of pure second-order logic:

$$\begin{aligned} \exists x \exists f {\mathsf {PA}}_{\mathsf {2}}[x, f]. \end{aligned}$$

Turning to the semantic side, one can think of the admissible structures of the theory ${\mathsf {PA}}_{\mathsf {2}}$ simply as those ${\mathcal {L}}_{A}$-structures that satisfy the axioms of ${\mathsf {PA}}_{\mathsf {2}}$. Since ${\mathcal {L}}_{A}$ contains no observation terms, no antecedent interpretation of such terms has to be considered. When applying the modal semantics of theoretical terms to Peano arithmetic, we obtain the following definition of the set of admissible structures:

### Definition 3

${\mathsf {PA}}_{\mathsf {2}}$**-admissible structures** $W_a$ designates the set of ${\mathcal {L}}_{A}$ structures that are admissible by way of an interpretation of the symbols in $V_t$ through the postulates ${\mathsf {PA}}_{\mathsf {2}}$. $Str(V_t)$ is the set of structures that interpret $V_t$ in whatever domain.

$$\begin{aligned} W_a ={\left\{ \begin{array}{ll} Mod(P_{TC}) \cap Str(V_t) \\ &{} \,\, \text {if }Mod(P_{TC}) \cap Str(V_t) \not = \emptyset \\ Str(V_t) &{} \,\, \text {if }Mod(P_{TC}) \cap Str(V_t) = \emptyset . \end{array}\right. } \end{aligned}$$

So, we must wonder whether the set $Mod(P_{TC}) \cap Str(V_t)$ is non-empty. This comes down to the question of whether or not the Peano axioms have a model. As is well known, it is impossible to prove the consistency of the Peano axioms using only finitistic methods. However, we have relative proofs of consistency, for example, by way of set-theoretic models of the Peano axioms, such as the van Neumann and the Zermelo numbers. If we accept such proofs of consistency, we can conclude:

$$\begin{aligned} W_a = Mod({\mathsf {PA}}_{\mathsf {2}}). \end{aligned}$$

Put differently, the class of admissible structures consists of those ${\mathcal {L}}_{A}$-structures of the form $\langle D, d, f \rangle$ (with domain *D*, a distinguished element $d \in D$, and a unary function $f \subseteq D^2$) that belong to the model class of ${\mathsf {PA}}_{\mathsf {2}}$. Notably, when defining the set of admissible structures in this way, we have lifted the requirement that there is a definite domain of interpretation for the theoretical terms.

How to think of the truth conditions of arithmetical statements? Given our specification of admissible structures, it should be clear that the modal semantics is also directly applicable to the case of Peano arithmetic. Consider any well-formed sentence in a definitional extension of language ${\mathcal {L}}_{A}$, for instance the sentence ‘$2 + 2 = 4$’. Since the addition symbol ‘+’ as well as the numerals are definable in ${\mathcal {L}}_{A}$, we could in principle give a translation of this sentence in ${\mathcal {L}}_{A}$. Let $\phi \in {\mathcal {L}}_{A}$ be the translation a well-formed sentence such as ‘$2 + 2 = 4$’. Note that $\phi$ will only contain the two primitive terms ‘0’ and ‘*S*’ as non-logical and thus theoretical terms.

According to the modal approach sketched above, we can say that an arithmetical sentence $\phi$ is true (relative to theory ${\mathsf {PA}}_{\mathsf {2}}$) if it is true in all admissible structures. Put differently, we say that $\phi$ is true if it is true in any structure in the model class $Mod({\mathsf {PA}}_{\mathsf {2}})$. To strengthen the analogy with the definition of theoretical truth described above, consider the corresponding Kripke frame: let ${\mathcal {M}}$ be a Kripke frame such that ${\mathcal {M}}=\langle W_a, R \rangle$ and $R = W_a \times W_a$. We saw that $W_a = Mod({\mathsf {PA}}_{\mathsf {2}})$. The accessibility relation *R* thus presents a total binary relation between any two structures satisfying ${\mathsf {PA}}_{\mathsf {2}}$.^5^ We can then express the assertion of a sentence $\phi \in {\mathcal {L}}_{A}$ (relative to theory ${\mathsf {PA}}_{\mathsf {2}}$) as follows:

$$\begin{aligned} {\mathcal {M}}\models \Box \phi . \end{aligned}$$

Equivalently, we can say that:

$$\begin{aligned} W_a \models _m \phi . \end{aligned}$$

which expresses that fact that $\phi$ is true if it is true in every structure of class $W_a$.

Given this general account of arithmetical truth, three comments should be made here. First, the modal semantics outlined allows us to capture the referential indeterminacy of mathematical terms. More generally, this semantics captures two types of indeterminacy. First, the referential indeterminacy of theoretical terms, including individual constants. Second, the semantic indeterminacy of theoretical sentences. A theoretical sentence is semantically indeterminate if and only if it is true in some admissible structure and false in some other structure that is also admissible. In the case of Peano arithmetic, we have referential indeterminacy of non-logical symbols, but not semantic indeterminacy of arithmetical sentences. Semantically indeterminate sentences of Peano arithmetic are ruled out by the fact that ${\mathsf {PA}}_{\mathsf {2}}$ is categorical and hence semantically complete.^6^

Second, the modal approach to arithmetic is in agreement with Carnap’s original discussion of the semantics of theoretical terms in Carnap (1939) and in later writings. Recall that Carnap’s central semantic thesis is that theoretical terms are interpreted indirectly through the postulates of a scientific theory. Thus, the meaning of theoretical terms is not specified directly in some metatheory as is the case with the observational terminology. Instead, such terms are implicitly defined by the postulates of a theory in which they occur. In the context of arithmetic, one can say analogously that the meaning of the non-logical primitive terms of ${\mathcal {L}}_{A}$ is specified only indirectly relative to the theory of Peano arithmetic. Put differently, the range of their possible semantic interpretations is specified by the theoretical constraints stated in the four axioms of ${\mathsf {PA}}_{\mathsf {2}}$.

Finally, the modal semantics nicely captures the structuralist view of arithmetical knowledge first introduced by Benacerraf.^7^ Specifically, we saw that Benacerraf’s central thesis concerns the rejection of a set-theoretic reductionism (of numbers to specific sets). A direct consequence of this diagnosis is the referential indeterminacy of arithmetical terms and the semantic indeterminacy of arithmetical discourse more generally. One can think of the theories of structuralism developed in response to Benacerraf’s thesis as different (and partly conflicting) attempts to deal with this kind of semantic indeterminacy in mathematics. Non-eliminative approaches such as Shapiro’s ante-rem structuralism propose to restore a face value semantics of arithmetical discourse by postulating abstract structures (and positions in them) as the true semantic relata of arithmetical terms and statements. Eliminative approaches, in turn, usually aim to represent the kind of indeterminacy of arithmetical terms based on a non-classical understanding of their semantics. The modal approach sketched above provides a semantics for arithmetical discourse that adequately captures this eliminative approach in structuralism.^8^

## Comparison with Hellman’s Modal Structuralism

The modal semantics for mathematical languages outlined above squares well with a non-realist (or eliminative) account of structuralism. It is therefore interesting to see how the approach relates to the most rigorously developed theory of eliminative structuralism, namely Hellman’s modal theory presented in Hellman (1989). In this section, we will compare the proposed semantic evaluation of arithmetical statements in terms of admissible structures with Hellman’s modal reconstruction of arithmetic.

As was mentioned in the introduction, Hellman’s theory was developed against the background of Putnam’s influential article “Mathematics without foundations” (1967) and the modal view of mathematics presented there. Putnam famously distinguishes between two “equivalent descriptions” of mathematical facts in his paper, namely “mathematics as set theory” on the one hand and “mathematics as modal logic” on the other. The former is essentially a realist or a Platonist position: it claims that all mathematical objects (such as numbers, groups, graphs, etc.) can be coded as structured sets in the standard set-theoretic universe. This position usually comes attached with a realist semantics for mathematical discourse. In particular, mathematical terms (such as the primitive terms of Peano arithmetic) are viewed to have fixed referents, namely particular sets. The truth conditions of mathematical statements, in turn, are also to be evaluated in terms of a set-theoretic semantics, by reference to particular models “living” in the universe of sets.

Putnam’s modal view, in contrast, presents a strictly nominalist account of mathematics. According to it, mathematical statements do not state facts about the objects of an abstract universe. Rather, they make conditional claims based on the assumption of the *possible* existence of such objects and of structures in which they occur. Hence, any theorem of number theory, analysis, or any other mathematical discipline can in principle be reformulated, in Putnam’s view, as a modalized conditional statement and thus “essentially a truth of pure modal logic” (Putnam 1967, p. 10).

We will turn to the details of the modal “if-thenist” reconstruction of mathematics below. Before doing so, it is important to stress that, according to Putnam, the modal account is mathematically equivalent to the more standard set-theoretical picture. This “object-modalities duality” is useful in two ways. In particular, one can make use of a set-theoretic and thus extensional semantics to further analyze the modal content of mathematical statements. Similarly, Putnam argues, one can make use of modalities in order to address problems inherent to the set-theoretic picture. This concerns, in particular, the issue of the indeterminacy of reference first raised by Benacerraf. Compare Putnam on this point in a remarkable passage:

($\ldots$) if one is puzzled by the question recently raised by Benacerraf: how numbers can be “objects” if they have *no* properties except order in a particular $\omega$-sequence, then, I believe, one can be helped by the answer: call them “objects” if you like ($\ldots$); but remember that these objects have the special property that each fact about them is, in an equivalent formulation, simply a fact about *any* $\omega$-sequence. “Numbers exist”; but all this comes to, for mathematics anyway, is that (1) $\omega$-sequences are *possible* (mathematically speaking); and (2) there are *necessary* truths of the form “if $\alpha$ is an $\omega$-sequence, then $\dots$” (whether any *concrete* example of an $\omega$-sequence exists or not). (ibid, pp. 11-12)

The view outlined here is essentially the modal-eliminative version of structuralism later elaborated in detail by Geoffrey Hellman (1989).

As Hellman points out in the book, any structuralist account of mathematics has to consist of three items, namely (i) a translation of a given mathematical discourse into a formal “structuralist language”, (ii) the specification of a “structuralist theory”, and finally, (iii) a “justification” of the proposed translation scheme (ibid, p. 7). Building on Putnam’s suggestions, Hellman develops a general modal structuralism that is applied to different mathematical theories. Specifically, his account is based on the interpretation of theories of pure mathematics in higher-order modal logic, in which the two modal operators (expressing “logico-mathematical” possibility and necessity) are treated as primitive. In what follows, we will discuss the three components of Hellman’s modal structuralism for the case of arithmetic, beginning with an exposition of his structuralist translation scheme.

The main aim in the first chapter of Hellman’s book is to provide a “modal-structural interpretation” of Peano arithmetic and analysis in a system of modal logic. This is done purely syntactically in terms of a translation scheme that allows one to transform any well-formed arithmetical statement into a purely “structuralist language”, namely a second-order language of modal logic. In Hellman’s view, the translation in question is warranted from a nominalistic point of view since it transforms ordinary arithmetical sentences (such as ‘$4 + 4 = 8$’) into modal statements that “say what would be the case in any (arbitrary) structure of the appropriate type without literally quantifying over any objects at all.” (ibid, p. 15)

Before turning to Hellman’s modal reconstruction of arithmetic in closer detail, it should be noted that his interpretation is based on a slightly different formalization of the Peano axioms than the one presented in the previous section.^9^ The signature of this arithmetical language, say ${\mathcal {L}}_{A'}$, contains three, not just two non-logical constants: a unary predicate ‘*N*’ (standing for the set of natural numbers), a binary relation symbol ‘*S*’ (for the successor relation), and the numeral ‘0’. Consequently, the axiomatization of second-order Peano arithmetic discussed by Hellman also differs from the above account to the effect that all quantifiers in the axioms are relativized to the domain predicate ‘*N*’.^10^ For instance, the axiom of induction now has the form

$$\begin{aligned} \forall X ([X(0) \wedge \forall y ((N(y) \wedge X(y)) \rightarrow X(S(y)))] \rightarrow \forall y (N(y) \rightarrow X(y))). \end{aligned}$$

Given this, Hellman’s modal reconstruction of arithmetical statements essentially consists of two components which he labels (i) the “categorical” and (ii) the “hypothetical” one. The hypothetical component consists in the translation of theorems of Peano arithmetic into boxed and universally quantified conditional sentences of the language of second-order modal logic. More specifically, given any theorem expressible in the language of ${\mathsf {PA}}_{\mathsf {2}}$ (or in a definitional extension thereof), say $\phi (N, 0, S) \in {\mathcal {L}}_{A'}$, its modal translate is given by the purely logical statement

$$\begin{aligned} \Box \forall X \forall x \forall f ({\mathsf {PA}}_{\mathsf {2}}(X, x, f) \rightarrow \phi (X, x, f)). \qquad \qquad \qquad {\mathrm{(HYP)}} \end{aligned}$$

Sentence HYP is formed by *universally Ramsifying* both the axioms of ${\mathsf {PA}}_{\mathsf {2}}$ and the theorem $\phi$, that is, by substituting all occurrences of the primitive terms ‘*N*’, ‘0’, and ‘*S*’ in these formulas by the universally quantified variables ‘*X*’, ‘*x*’, and ‘*f*’ respectively.

According to Hellman, HYP can be taken to express the “modal-structural content” of the arithmetical sentence $\phi$. This implicit structuralist assertion made explicit in HYP is characterized informally as follows:

($\ldots$) we should like to construe a (pure) number-theoretic statement as elliptical for a statement as to what would be the case in any structure of the appropriate type. In this case, the structures are, of course, “progressions” or “$\omega$-sequences” ($\ldots$). (ibid., p. 16)

Put differently, the structural content of statement $\phi$ can be paraphrased as follows: to assert $\phi$ means to assert that it is necessarily true or true in any $\omega$-sequence satisfying the axioms of second-order Peano arithmetic.

Let us consider a simple example to further illustrate the account: the statement “$2 + 2 = 4$” is a basic arithmetical theorem that is derivable from the axioms of Peano arithmetic. The statement can easily be expressed in the extension of our basic language by an addition sign.^11^ Let $\varphi$ be the corresponding statement “$S(S(0)) + S(S(0)) = S(S(S(S(0))))$” in ${\mathcal {L}}_{A'} \cup \{ +\}$. According to Hellman’s translation manual, the structural content implicit in theorem “2 + 2 = 4” is expressed in the following complex modal sentence:

$$\begin{aligned} \Box \forall X \forall x \forall f \forall g [{\mathsf {PA}}_{\mathsf {2}}^{*}(X, x, f, g) \rightarrow g(f(f(x)), f(f(x))) = f(f(f(f(x))))]. \end{aligned}$$

Note that the translation manual (HYP) delivers a purely logical statement of the underlying “structuralist language”, i.e. a second-order modal language with relation and function variables (of each arity) and corresponding quantifiers.

Moreover, while Hellman’s interpretation of arithmetical statements in modal logic is purely syntactic in character, his underlying motivation is a semantic one. What the modal translation of “$2 + 2 = 4$” expresses or asserts is essentially a metatheoretic claim, namely that a certain arithmetical fact (that 2 plus 2 equals 4) applies to *any* number system satisfying the Peano axioms. Thus, HYP is to be understood as an object-linguistic formulation (in Hellman’ structuralist language) of the ”metalinguistic” claim that

$$\begin{aligned} \Box \forall {\mathcal {M}} ({\mathcal {M}} \models {\mathsf {PA}}_{\mathsf {2}} \Rightarrow {\mathcal {M}} \models \phi ). \end{aligned}$$

This alternative expression of the structural content of an arithmetical statement $\phi$ is clearly metamathematical in Hellman’s own words since it makes use of the metatheoretic concepts of the quantification over models and of the truth in a model (see, ibid, p. 18).^12^ Hellman’s reformulation of this model-theoretic statement in terms of the purely object-mathematical statement HYP is clearly motivated by his nominalism. Nevertheless, the content expressed by HYP is genuinely semantic or model-theoretic in character.

Let us now turn to the second, “categorical” component of the modal reconstruction. As Hellman points out, the modal-structuralist interpretation of number theory is based on the assumption that “$\omega$-sequences are possible” (in the sense of logical or mathematical possibility). Without this additional assumption, the modal if-thenist reconstruction of arithmetical statements expressed in HYP would be subject to a “vacuity problem” first addressed against traditional (i.e. non-modal) forms of if-thenism.

Roughly put, if it were impossible that $\omega$-sequences exist, then the antecedent in HYP would turn out false. Given the principle of *ex falso quodlibet*, it would follow that the modal translation of any statement expressible in the language of arithmetic would turn out to be true. Thus, the meaningfulness of HYP as a structuralist reconstruction of arithmetical statements presupposes that there possibly exists a model of ${\mathsf {PA}}_{\mathsf {2}}$. This modal existence assumption can be expressed in terms of the following statement (again in the second-order language of modal logic):

$$\begin{aligned} \lozenge \exists X \exists x \exists f ({\mathsf {PA}}_{\mathsf {2}}(X, x, f)). \qquad \qquad \qquad {\mathrm{(CAT)}} \end{aligned}$$

Note that CAT is essentially a modalized version of the Ramsey sentence of Peano arithmetic outlined in Sect. 3. It is used as an existence postulate in Hellman’s theory stating the possible existence of ${\mathsf {PA}}_{\mathsf {2}}$- structures.

Given this brief outline of Hellman’s account, how does it compare to the modal semantics outlined in the previous section? As we saw, both accounts provide a logical analysis of arithmetical knowledge in line with eliminative structuralism. That said, there are several differences between the two approaches. Arguably the central difference concerns the very notion of modal operators. Andreas’ modal approach to theoretical terms is, as we saw, essentially model-theoretic in character. It provides a Kripke frame semantics for theoretical statements based on the concept of admissible structures. Hellman’s “modal-structuralist interpretation” of arithmetic, in contrast, is syntactic in character (even though also motivated by semantic considerations). His reconstruction consists of a translation manual that transforms arithmetical statements into complex sentences of second-order language with modal operators. It is crucial to emphasize that these operators—or the concepts of “logico-mathematical” possibility and necessity expressed by them—are treated as primitive by Hellman. Thus, unlike in Andreas’ modal semantics, the very idea of describing the logical behaviour of these operators in terms of a set-theoretic semantics is deliberately ruled out by him. The reason for this is Hellman’s mathematical nominalism: providing a Kripke semantics for modal expressions would effectively reduce the “modal description” of mathematics to a set-theoretical and thus to a realist one. Compare Hellman on this point, commenting on Putnam’s modalism in mathematics:

The *locus classicus* of [the modal] approach is Putnam’s ‘Mathematics without Foundations’, in which it was suggested that many of the problems plaguing objects-platonism (and, in particular, the identification of mathematics with set theory) could be overcome by reinterpreting mathematics, as standardly presented, in a modal language, in which a notion of mathematical or logical possibility is taken as primitive. Since the work of Kripke, we have become familiar with the procedure of providing a set-theoretic semantics for the operators of modal logic. What Putnam proposed was the opposite procedure of working with those operators as primitive, and using them to reconstruct Platonist discourse in such a way that literal quantification over abstract objects could be made to disappear entirely. (ibid. pp. 7-8)

Thus, following Putnam’s original suggestion, the modal operators in Hellman’s structuralist language are also specified purely syntactically, based on an S5 logical system. Hellman’s item (ii) of his list, namely a “structuralist theory” used for the specification of the structural content of arithmetical statements, is thus an S5 second-order modal logic.^13^

The uses of modal logic therefore differ significantly in the two accounts: for Hellman, the modal-logical reconstruction of arithmetic serves primarily the aim of nominalization, that is, of getting rid of any commitment to the actual existence of numbers and number sequences. In the reconstruction of arithmetic based on Andreas’ modal approach to theoretical terms, modal logic is used primarily to represent the referential indeterminacy of the arithmetical vocabulary. The reference to abstract objects such as sets is viewed as permissible for the interpretation of the primitive symbols of Peano arithmetic.

This observation leads to a second difference, namely on how precisely the structural content of arithmetic is characterized in the two accounts. Structuralism is essentially viewed as a semantic thesis in our modal approach: as we saw, the structural content expressed by an arithmetical statement can be analyzed in terms of its truth conditions specified relative to a class of theoretically admissible structures. In contrast, in Hellman’s approach, the modal component plays a surprisingly small role in his structuralist reconstruction of arithmetic. Recall that Hellman’s theory is usually characterized as a form of eliminative structuralism, that is, an account that understands talk about mathematical structures as a kind of shorthand for talk about all models of a theory. Thus, eliminative theories of structuralism do not assume the existence of abstract structures in addition to more concrete mathematical systems or models.^14^

What makes Hellman’s modal theory a form of eliminative structuralism in this sense is the universal Ramsification and the “if-thenist” reconstruction of mathematical statements outlined above. More specifically, it is the suggestion to translate any statement of arithmetic $\phi$ into a universally quantified conditional statement of the form:

$$\begin{aligned} \forall X \forall x \forall f ({\mathsf {PA}}_{\mathsf {2}}(X, x, f) \rightarrow \phi (X, x, f)). \qquad \qquad \qquad \qquad (\text {HYP}^{-}) \end{aligned}$$

Arguably, it is this translation (without the box operator) that expresses the structural content of $\phi$, namely the fact that $\phi$ is true in any model of theory ${\mathsf {PA}}_{\mathsf {2}}$. Prefixing the statement with a primitive necessity operator (as is suggested in HYP) has the additional function to rule out any ontological commitments that come with the universal generalization over the models of a given theory. Thus, while the if-thenist manoeuvre itself leads to a form of eliminative structuralism, the modal component serves the purpose of ruling out any ontological commitments that come with the assertion of an arithmetical statement under a set-theoretical interpretation.

Notice, however, that if the structuralist thesis in Hellman’s account is viewed in this way, the two approaches actually turn out to be quite similar. Hellman’s HYP essentially expresses the generalization over all structures admissible for the semantic interpretation of an arithmetical statement. Theoretical admissibility is expressed in the object language here, namely in terms of the Ramsified version of the Peano axioms formulated in the antecedent of HYP.  In our proposed semantics for the arithmetical language, one also generalizes over the admissible structures of an arithmetical statement. However, we have defined the notion of an admissible structure metatheoretically and used it in the formulation of a suitable Kripke semantics.

This similarity between the two approaches becomes more evident if one accepts Hellman’s modalized version HYP, but evaluates it semantically by use of Kripke models. We mentioned above that, according to Hellman, a central component of any structuralist interpretation of mathematical theories consist in the “justification” of the suggested translation manual. (This is the third component of his structuralist account of mathematics.) In the case of his modal theory, this justification consists in showing the accuracy and adequacy of the translation scheme HYP.  More precisely, it is to show that any statement $\phi$ of the language of Peano arithmetic under a “literal Platonist reading” and its modal translate $\phi _{\text {msi}}$ “are fully equivalent for mathematical purposes” (ibid, p. 33). Mathematical equivalence is understood here as the fact that the truth of the statement is preserved by the modal translation. Thus, justifying the translation scheme for Hellman means to show that $\phi _{\text {msi}}$ holds if and only if $\phi$ holds under a Platonist interpretation. Platonism here just means that the mathematical language is interpreted in a given domain of abstract objects, namely the universe of sets.

In the first chapter of his book, Hellman outlines two ways to justify this equivalence for the case of Peano arithmetic: an “external” one which allows one to draw to the resources of set-theory as well as a “justification from within” his nominalist modal framework. We leave aside here the latter approach which centers on the notion of internal categoricity. The external, set-theoretic justification, in turn, is based on the specification of two semantic frameworks—namely a realist notion of ”truth in the standard model” and the modal-structuralist notion of ”truth in any possible model”—that allows one to compare an arithmetical statement and its modal translate (ibid., pp. 34-38). Specifically, Hellman sketches a proof of the following equivalence result in this context: a statement $\phi$ expressed in the second-order language of Peano arithmetic is true in the standard model ${\mathcal {M}}$ of ${\mathsf {PA}}_{\mathsf {2}}$ if and only if $\phi _{\text {msi}}$ is a logical truth of modal logic. This is done by first showing that the translation scheme $\text {HYP}^{-}$ without the box operator is bivalent and truth-preserving: if $\phi$ is true in the standard model ${\mathcal {M}}$, then the if-thenist translation $\phi _{\text {msi}}^{-}$ is a truth of pure second-order logic.

Second, turning to the modal component of his reconstruction, Hellman follows Putnam in that “the Platonist may make reasonable sense of the modality in question by providing a set-theoretic semantics for it ($\ldots$)” (ibid, p. 36). In particular, by drawing on work by Cocchiarella (1975), Hellman suggests to think of the semantic interpretation of modal translations of arithmetical statements in terms of “model structures” consisting of a fixed domain of individuals, model-theoretic structures of a given type as the worlds, and a universal accessibility relation that connects any two number systems. Given this framework, the mathematical adequacy of the modal translation of a sentence $\phi$ is justified in terms of the following “Platonist *equivalence theorem*” (ibid., p. 37):^15^

$$\begin{aligned} {\mathsf {PA}}_{\mathsf {2}} \text { logically implies } \phi \text { iff } \phi _{\text {msi}} \text { is a logical truth.} \end{aligned}$$

This result can be used to show the semantical equivalence between our proposed modal structuralism and Hellman’s approach. To see this, let us compare how the relational structures are related in the two accounts. Recall that in Andreas’ modal semantics, the relevant structures are Kripke frames of the form ${\mathcal {M}} = \langle W_a, R \rangle$, where $W_a$ stands for the class of admissible structures relative to theory ${\mathsf {PA}}_{\mathsf {2}}$ and language ${\mathcal {L}}_A$. Given how the class of admissible structures was defined in Sect. 4, we saw that $W_a$ forms the model class of theory ${\mathsf {PA}}_{\mathsf {2}}$. In turn, one can think of the structure described by Hellman’s S5 semantics as a Kripke frame of the form ${\mathcal {N}} = \langle D, Str(V_{t}), R \rangle$, where *D* is a universal domain of individuals, $Str(V_{t})$ the class of all ${\mathcal {L}}_{A}$ structures, and *R* a universal accessibility relation. Given this set-up, it can easily be shown that the two accounts are semantically equivalent. Thus, for any statement $\phi \in {\mathcal {L}}_{A}$, we have:

$$\begin{aligned} {\mathcal {M}} \models \Box \phi \text { if and only if } {\mathcal {N}} \models \phi _{\text {msi}}. \end{aligned}$$

Assume first that ${\mathcal {N}} \models \phi _{\text {msi}}$. It follows that the $\Box$ operator in $\phi _{\text {msi}}$ ranges over all ${\mathcal {L}}_{A}$-structures. Moreover, the antecedent in $\phi _{\text {msi}}$ effectively relativizes the universal quantifiers to range over interpretations of the primitive terms ‘*N*’, ‘0’, and ‘*S*’ in the models of ${\mathsf {PA}}_{\mathsf {2}}$. Thus, $\phi _{\text {msi}}$ expresses that in every ${\mathcal {L}}_{A}$-structure in which the axioms of ${\mathsf {PA}}_{\mathsf {2}}$ are true, $\phi$ must be true as well. This is exactly what ${\mathcal {M}} \models \Box \phi$ states, given that $W_a = Mod({\mathsf {PA}}_{\mathsf {2}})$. Second, assume that ${\mathcal {M}} \models \Box \phi$. Hence, $\phi$ is true in all models of ${\mathsf {PA}}_{\mathsf {2}}$. Consequently, the non-modal conditional statement $\phi _{\text {msi}}^{-}$ is true in every ${\mathcal {L}}_{A}$-structure. It follows that $\phi _{\text {msi}}$ is true in ${\mathcal {N}}$. Given this result, it becomes clear that, at least once a Kripke reading of the modal operators in HYP and CAT is adopted, Hellman’s modal structuralism turns out to be equivalent with the modal structuralism derived here from a Carnapian semantics of theoretical terms. However, if we follow Hellman and take the model operators in HYP and CAT as primitive, the two variants of modal structuralism remain semantically incomparable.

Philosophy is competitive, so it is tempting to make a case for one variant of modal structuralism against the other. It is difficult to resist this temptation. However, we should note that the two variants of modal structuralism score differently in different dimensions. So, if there is a winner, this victory holds only with respect to certain standards of evaluation. In what follows, we will try to identify benefits and shortcomings concerning the three components of a structuralist account of mathematics: (i) the translation scheme into a structuralist language, (ii) the structuralist theory, and (iii) the justification of the proposed translation scheme.

What are the benefits of Hellman’s modal structuralism compared to ours? Arguably, both Hellman’s and our approach work out the components of a structuralist account of mathematics in a coherent manner. The proposals for (ii) and (iii) even exhibit substantial commonalities. However, the nominalism of Hellman’s approach seems to be a key benefit compared to ours. We agree with Hellman, Putnam and Ockham that it is desirable to work with fewer entities if there is a way to do so. Otherwise, we find it difficult to identify clear-cut advantages of Hellman’s structuralism over the ours.

What are the benefits of the proposed modal structuralism compared to Hellman’s? First, the translation scheme into a structuralist language is much simpler. Once an arithmetical statement has been translated into the language of second-order Peano arithmetic, we merely need to prefix the translation with a modal box operator. Unlike Hellman’s translation scheme, no second-order variables or additional quantifiers are needed. Hence, our modal structuralism stays closer to the surface grammar of mathematical discourse than Hellman’s. Relatedly, the simplicity of the translation scheme is a virtue in light of standard criteria for assessing theories. If two competing theories capture the same subject matter, we should prefer the simpler one unless the other theory scores better in other dimensions. There is wide-ranging consensus on this being a reasonable criterion for the evaluation of theories in the sciences, mathematics, and philosophy.

Another benefit we see in our modal structuralism is that modal operators are not left without an explanation. Such operators are rather understood in the standard way. This means that our modal structuralism coheres better with our understanding of the modal operators in a number of different areas, including modal logic as taught in an advanced course to non-classical logic. Moreover, we take it almost for granted that it is a virtue of any account in philosophy not to use highly abstract notions, such as the modalities, without an explanation. This is simply part of the game called *analytic philosophy*, as initiated by Frege, Russell, Carnap, and others. Other types of doing philosophy may be worth pursuing too, but we are not going to do this in this paper. This is not say that Hellman’s account of structuralism parts from the analytic tradition, but to argue that our modal structuralism follows this tradition more strictly.

In sum, Hellman’s modal structuralism has the benefit of achieving a reduction of entities. No sets are needed in his account. Our modal structuralism has the benefit of a simpler translation scheme into a structuralist language, which comes with the benefit of staying closer to the surface grammar of arithmetical discourse. The latter benefit may or may not be argued to be additional to the former; we do not take a side here. Further, our modal structuralism does not take the modal operators as primitive. Since these operators are understood in the standard way, our account of structuralism coheres better with the understanding of modality in many areas of contemporary philosophy and philosophical logic. Drawing on standard criteria for assessing scientific theories, we can therefore say that our modal structuralism exhibits a higher degree of *external coherence*. To finally evaluate the two accounts, let us assume that the following criteria have equal weights: (i) avoiding unnecessary entities, (ii) simplicity, (iii) external coherence, and (iv) the criterion of avoiding primitive notions which are abstract. Then our modal structuralism scores better than Hellman’s. In this sense, we have reason to prefer the former over the latter.

## Arithmetical Truth

Andreas’ modal semantics applied to arithmetic yields the following understanding of arithmetical truth: a statement $\phi$ of Peano arithmetic is true if and only if $\phi$ is true in all models of the Peano axioms, provided there is one. Likewise for definitional extensions of Peano arithmetic. Moreover, we can apply the modal semantics to whatever axiomatic theory in mathematics. What is the philosophical interest of such an explication of arithmetical and mathematical truth? Recall that we have motivated the modal semantics by a deviation from logical atomism in the introduction. Let us now try to characterize the philosophy of logical atomism by distinct semantic doctrines.

Suppose ${\mathcal {L}}$ is a language that is properly meaningful according to the philosophy of logical atomism. Then, first, ${\mathcal {L}}$ must have a well defined and definite domain of interpretation. Second, every non-logical symbol of ${\mathcal {L}}$ must have a well defined and definite extension. It is less obvious whether we should characterize the semantics of logical atomism by the requirement that, for every object of *D*, there is a closed individual term of ${\mathcal {L}}$ that refers to this object. This semantic doctrine could be motivated by the substitutional semantics of the quantifiers sketched in Wittgenstein’s *Tractatus* (1922). This brief characterization of logical atomism is of course not intended to be historically accurate in the sense that the philosophers of logical atomism explicitly and literally maintained said semantic doctrines. We merely try to characterize the semantic views of Frege, Russell, and Wittgenstein using the language of model theory, even though none of these had a fully fledged model theory at his disposal.

It is now almost trivial to observe that the semantic doctrines of logical atomism are lifted by the modal semantics recommended in the present paper. For example, there is no need to specify a unique interpretation of the numerals of Peano language. Relatedly, we do not have to specify a unique interpretation of the successor function. We merely need to be able to specify some interpretation of the numerals and the successor function such that all Peano axioms are satisfied by this interpretation. The proponent of an eliminative structuralism in mathematics explicitly rejects the semantic doctrines of logical atomism. This is clearly also the case with Hellman’s modal structuralism and the if-thenist reconstruction of mathematics underlying it. Nevertheless, while both Hellman’s and Andreas’ accounts agree in their opposition to an atomistic understanding of mathematical languages, their philosophical motivations for this differ. In Hellman’s case, semantic atomism is ruled out by his nominalism. Andreas’ account, as we saw, is primarily viewed as an alternative to the atomist’s premise of the uniqueness of reference.

The semantics of logical atomism seems to be motivated by the idea that there is a definite, uniquely determined, and actual world to which our language refers to. No such assumption is implied by Andreas’ modal semantics. Quite to the contrary, this semantics invites us to take a multitude of possibilities as representation of certain mathematical entities, such as the natural numbers. None of the possibilities is considered closer to reality than another. In this modal representation of mathematical entities, we take those sentences to be true that are true in each possible world. Since the set of possible worlds is sententially characterized, it is not even necessary to know the possible worlds precisely in order to figure out which sentences of the respective language are true. We can derive what is true from the set of sentences that determines the set of possible worlds. For example, when doing calculations in arithmetic, there is no need to consider all the possible interpretations of the numeral 7, which include a Zermelo number, a van-Neumann number, the numeral 7 itself, and many more.

Taking a set of possibilities as representation of certain mathematical entities may be seen as the major philosophical proposal of the modal semantics. There is no need to interpret mathematical language in a rigid and unique manner. Sometimes it is interesting and valuable to further constrain the set of possibilities determined by an axiomatic theory, sometimes it is not. In the case of the Peano axioms, it seems as if we can embrace the different possible interpretations as they are. Consequently, we consider only those arithmetical statements true that are true in all interpretations which satisfy the Peano axioms. The modal semantics, thus, explicates an understanding of truth that fits eliminative structuralism in mathematics.

This understanding of truth and reality has an interesting connection to modal logics of belief and knowledge, first devised by Hintikka (1962). Hintikka’s central idea is to characterize the belief set of an epistemic agent by a set *W* of possible worlds: each member of the set is considered a possibility by the agent. Possible worlds outside of the set *W* are not considered possible. Then, he introduces a belief operator ${\mathbf {B}}$ in the style of a modal operator with an S5 semantics. ${\mathbf {B}} \phi$—in words: the agent believes $\phi$—is true if and only if $\phi$ is true in all members of *W*. In multi-agent systems, different belief sets are characterized by different modal operators accordingly. Together with AGM belief revision theory by Alchourrón et al. (1985) and Gärdenfors (1988), Hintikka’s work has given rise to a new research area, called *dynamic epistemic logic*.^16^

Drawing on these ideas from epistemic logics, we can say that the Peano axioms determine and encapsulate our beliefs about what the natural numbers are. They fall short of determining unique referents of the numerals, though. According to eliminative structuralism, there is no need to look for further constraints on the interpretation of arithmetical language. Frege and Russell, by contrast, were looking for such constraints so as to find unique referents of numerals. Not surprisingly, Frege insisted on sharply distinguishing between axioms and definitions in the controversy with Hilbert.^17^

## Conclusion

The aim in this article was to connect recent work on the semantics of theoretical terms (inspired by Carnap’s notion of indirect or incomplete interpretation) with a structuralist account of arithmetic. Specifically, we applied Andreas’ modal semantics for theoretical statements of scientific theories to the semantic evaluation of statements of Peano arithmetic. As was shown, the resulting modal semantics squares well with a particular structuralist account of arithmetic, namely, eliminative structuralism. This was characterized as the view that the structural character of arithmetical knowledge is best expressed in terms of the generalization over all possible interpretations of Peano arithmetic. We saw that the central semantic consequence of this view, namely the indeterminacy of reference of arithmetical terms, is adequately modeled in the modal approach. Given this, we then compared the modal semantics for arithmetic to the most rigorously developed theory of eliminative structuralism, namely Hellman’s modal structuralism. As was shown, there are several interesting points of contact between the semantic view and Hellman’s account. Using standard criteria for assessing theories in science and mathematics, we have argued that our proposed modal structuralism scores better than Hellman’s. However, our proposal fails to meet the nominalist tenets of Hellman’s structuralism.

At the end of his seminal “What Numbers Could not Be” (1965), Benacerraf points out the benefits of a structuralist conception of natural numbers when it comes to learning the language of arithmetic. On this conception, we are free to take number words as numbers since the crucial properties of a specific natural number are determined by its relations to other members in the sequence. The existence of different systems of numerals is not a problem for such an interpretation of arithmetic once we have realized that the search for unique referents of arithmetical expressions is futile. Put in a nutshell, referential indeterminacy allows for flexibility that is crucial for learning and applying arithmetic. This is in line with an attempt by Stegmüller (1970) to show that incompletely interpreted theoretical terms allow for flexibility that is crucial for further developments of extant scientific theories. Insisting on a complete interpretation of the technical terms in a scientific theory would block scientific progress. He termed this consideration the *Braithwaite-Ramsey conjecture*. This conjecture has recently and independently been formulated by Halvorson (2019, p. 282). We leave for future research a precise formulation of the conjecture in question and its application to cognitive aspects of arithmetic.

## Footnotes

1 Ramsey calls the T-postulates *axioms* and the set of C-postulates *dictionary*. For simplicity, we follow Carnap’s terminology.

2 The former problem is addressed in Carnap (1961), where he uses Hilbert’s $\epsilon$-operator for expressing that the Carnap sentence yields only an indefinite description of theoretical terms. For a translation of the present semantics of theoretical terms into the language of the epsilon operator, see Andreas and Schiemer (2016).

3 Andreas (2010) assumes a fixed domain of interpretation for the set $V_t$ of theoretical terms. This assumption, however, is not needed for the modal semantics to work. Theoretical and mathematical entities of the theory in question may well be characterized by the postulates $P_{TC}$. Hence, there is no need to specify a fixed domain of interpretation in the metalanguage.

4 We adopt the semantic characterization of a definition from Beth’s definability theorem. This theorem says that any implicit definition can be transformed into an explicit definition. But the notion of an implicit definition may not concern us here. We deliberately avoid using this notion because it has been used and understood in a number of different ways.

5 Given that fact the theory ${\mathsf {PA}}_{\mathsf {2}}$ is categorical, which means that any two of its models are isomorphic, one can think of *R* as representing the isomorphism relation $\cong$ on $Mod({\mathsf {PA}}_{\mathsf {2}})$.

6 See Button and Walsh (2018) for a proof of this result.

7 It should be noted here that while Benacerraf’s 1965 paper is usually considered to be the starting point of the modern debate on mathematical structuralism, structuralist ideas in fact have a long history, tracing back to work nineteenth-century mathematics. In the case of arithmetic, Dedekind’s famous monograph *Was sind und was sollen die Zahlen* of 1888 should be mentioned as an early and decidedly structuralist contribution. See, in particular, Reck and Schiemer (2000) for further details on the prehistory of structuralism.

8 Other non-classical semantics of arithmetical statements have been discussed in the literature that are also in line with eliminative structuralism. See, e.g., the choice-semantics for mathematical terms outlined in Schiemer and Gratzl (2016) or a Ramsey semantics developed recently in Leitgeb (2020). For the equivalence of the choice semantics and the modal approach discussed in the present paper, see Andreas and Schiemer (2016).

9 In order to make Hellman’s account better comparable to our approach, we give a slightly modified presentation of the logical details of his modal reconstruction of arithmetic here.

10 Compare also Reck and Price (2000) on this point.

11 As is well-known, we can introduce the addition sign ‘+’ to our primitive arithmetical vocabulary in terms of a recursive definition, i.e., by the following two clauses: (i) $n +0 = n$ and (ii) $n + S(m) = S(n + m)$. Let ${\mathsf {PA}}_{\mathsf {2}}^{*}$ be the theoretical extension of ${\mathsf {PA}}_{\mathsf {2}}$ by these two recursive clauses. Moreover, any numeral can be explicitly defined in terms of the sign ‘0’ and ‘*S*’, e.g. “$2 = S(S(0))$”.

12 Compare this metatheoretic analysis of the content of statement “$2 + 2 = 4$” with Andreas’ modal analysis given in terms of admissible structures in Sect. 4. A closer discussion of the differences between the accounts is given below.

13 Compare again Hellman on this point: “($\ldots$) the background modal logic must not contain the Barcan formula. (As to what else it should contain, the natural choice will be S-5 since we are concerned here with an absolute, mathematico-logical sense of possibility.” (ibid. p. 17).

14 Compare, e.g., Shapiro (1997) and Reck and Schiemer (2020) on the distinction between eliminative and non-eliminative structuralism.

15 It should be noted that Hellman does not provide precise definitions of the semantic concepts of second-order logical consequence and modal logical truth, nor does he state a proof of the equivalence result.

16 See van Ditmarsch et al. (2008) for an accessible introduction.

17 See, e.g., Frege’s letter to Hilbert of 27-th of December 1899 in Frege (1980).

## References

[CR1] Alchourrón MA, Gärdenfors P, Makinson D (1985). On the logic of theory change: Partial meet contraction functions and their associated revision functions. Journal of Symbolic Logic.

[CR2] Andreas H (2010). A modal view of the semantics of theoretical sentences. Synthese.

[CR3] Andreas H, Schiemer G (2016). A choice-semantical approach to theoretical truth. Studies in History and Philosophy of Science Part A.

[CR4] Balzer W, Moulines CU, Sneed J (1987). An architectonic for science. The structuralist program.

[CR5] Benacerraf P (1965). What numbers could not be. The Philosophical Review.

[CR6] Button T, Walsh S (2018). Philosophy and model theory.

[CR7] Carnap R (1928). Der Logische Aufbau der Welt.

[CR8] Carnap, R. (1936/37). Testability and meaning. *Philosophy of Science**3/4*:419–471/1–40.

[CR9] Carnap R (1939). Foundations of logic and mathematics.

[CR10] Carnap R (1952). Meaning postulates. Philosophical Studies.

[CR11] Carnap R (1958). Beobachtungssprache und theoretische Sprache. Dialectica.

[CR12] Carnap, R. (1961). On the use of Hilbert’s $$\epsilon $$-operator in scientific theories. In Y. B.-H. et al. (Ed.), *Essays on the foundations of mathematics* (pp. 156–164). Jerusalem: The Magnus Press.

[CR13] Carnap R (1966). Philosophical foundations of physics.

[CR14] Cocchiarella NB (1975). On the primary and secondary semantics of logical necessity. Journal of Philosophical Logic.

[CR15] Frege G, Gabriel G, Hermes H, Kambartel F, Thiel C, Veraart A, McGuinness B, Kaal H (1980). Frege to Hilbert, December 27, 1899. Philosophical and mathematical correspondence.

[CR16] Gärdenfors P (1988). Knowledge in flux.

[CR17] Halvorson H (2019). The logic in philosophy of science.

[CR18] Hellman G (1989). Mathematics without numbers: towards a modal-structural interpretation.

[CR19] Hintikka J (1962). Knowledge and belief.

[CR20] Leitgeb, H. (2020). Ramsification and semantic indeterminacy. *Unpublished manuscript* .

[CR21] Lewis D (1970). How to define theoretical terms. Journal of Philosophy.

[CR22] Putnam H (1967). Mathematics without foundations. The Journal of Philosophy.

[CR23] Ramsey FP, Braithwaite RB (1950). Theories. The foundations of mathematics and other logical essays.

[CR24] Reck E, Price M (2000). Structures and structuralism in contemporary philosophy of mathematics. Synthese.

[CR25] Reck E, Schiemer G (2000). The prehistory of mathematical structuralism.

[CR26] Reck, E., & Schiemer, G. (2020). Structuralism in the philosophy of mathematics. In E. N. Zalta (Ed.), *The Stanford Encyclopedia of Philosophy* (Spring 2020 edn). Metaphysics Research Lab, Stanford University.

[CR27] Russell B (2010). The philosophy of logical atomism.

[CR28] Schiemer G, Gratzl N (2016). The epsilon-reconstruction of theories and scientific structuralism. Erkenntnis.

[CR29] Shapiro S (1997). Philosophy of mathematics: Structure and ontology.

[CR30] Sneed J (1979). The logical structure of mathematical physics.

[CR31] Stegmüller, W. (1970). *Probleme und Resultate der Wissenschaftstheorie und Analytischen Philosophie. Bd. 2 - Theorie und Erfahrung, Teilbd. 1*. Berlin-Heidelberg-New York: Springer.

[CR32] van Ditmarsch H, van der Hoek W, Kooi B (2008). Dynamic epistemic logic.

[CR33] Whitehead AN, Russell B (1925). Principia Mathematica.

[CR34] Wittgenstein L (1922). Tractatus Logico-Philosophicus.

